# Supplementary material for: From sequence to enzyme mechanism using multi-label machine learning
Source: BMC Bioinformatics. 2014 May 19;15:150. doi: 10.1186/1471-2105-15-150 (PMC4229970; doi:10.1186/1471-2105-15-150)
Supplement: Additional file 2 — Java code of ml2db. Additional file ml2db_code.tar.gz contains the Java source code to run the multi-label machine learning experiments and save the results to database. The code’s Javadoc is included. [file 1471-2105-15-150-S2.zip › additional file 2/ml2db/utils/doc/edu/cornell/lassp/houle/RngPack/RandomElement.html]

RandomElement


JavaScript is disabled on your browser.


- Overview
- Package
- Class
- Use
- Tree
- Deprecated
- Index
- Help

- Prev Class
- Next Class

- Frames
- No Frames

- All Classes

- Summary:
- Nested |
- Field |
- Constr |
- Method

- Detail:
- Field |
- Constr |
- Method


edu.cornell.lassp.houle.RngPack

## Class RandomElement

- java.lang.Object
- - cern.colt.PersistentObject
  - - edu.cornell.lassp.houle.RngPack.RandomElement

- All Implemented Interfaces:
  :   java.io.Serializable, java.lang.Cloneable

  Direct Known Subclasses:
  :   RandomJava, RandomSeedable, RandomShuffle

  ---

    

  ```
  public abstract class RandomElement
  extends PersistentObject
  implements java.lang.Cloneable
  ```

  RandomElement is an abstract class that encapsulates uniform random number
  generators.

  Subclasses have entirelly deterministic behaviour: Constructing two instances
  with the same parameters at any two distinct points in time will produce
  identical sequences of numbers.

  To base a class on it, you must define the method `raw()` as
  described below. It is also likely that you will want to define a constructor
  or another mechanism for seeding the the generator. The other classes defined
  in `RandomElement` add value to the numbers generated by
  `raw()`

  Source
  code  is available.

  Version:
  :   1.0

  Author:
  :   Paul Houle  (E-mail:
      houle@msc.cornell.edu)

  See Also:
  :   `RandomJava`,
      `RandomShuffle`,
      Serialized Form

- - ### Constructor Summary

    Constructors

    Constructor and Description

    `RandomElement()`
  - ### Method Summary

    Methods

    | Modifier and Type | Method and Description |
    | --- | --- |
    | `int` | `choose(int hi)` |
    | `int` | `choose(int lo, int hi)` |
    | `double` | `gaussian()` gaussian() uses the Box-Muller algorithm to transform raw()'s into gaussian deviates. |
    | `double` | `gaussian(double sd)` |
    | `double` | `powlaw(double alpha, double cut)` generate a power-law distribution with exponent `alpha` and lower cutoff `cut` |
    | `abstract double` | `raw()` Returns a uniformly distributed pseudo-random number. |
    | `void` | `raw(double[] d)` Fill an entire array with doubles. |
    | `void` | `raw(double[] d, int n)` Fill part or all of an array with doubles. |
    | `double` | `uniform(double lo, double hi)` |

    - ### Methods inherited from class cern.colt.PersistentObject

      `clone`
    - ### Methods inherited from class java.lang.Object

      `equals, getClass, hashCode, notify, notifyAll, toString, wait, wait, wait`

- - ### Constructor Detail


    - #### RandomElement

      ```
      public RandomElement()
      ```
  - ### Method Detail


    - #### choose

      ```
      public int choose(int hi)
      ```

      Parameters:
      :   `hi` - upper limit of range

      Returns:
      :   a random integer in the range 1,2,... ,**hi**


    - #### choose

      ```
      public int choose(int lo,
               int hi)
      ```

      Parameters:
      :   `lo` - lower limit of range
      :   `hi` - upper limit of range

      Returns:
      :   a random integer in the range **lo**,
          **lo**+1, ... ,**hi**


    - #### gaussian

      ```
      public double gaussian()
      ```

      gaussian() uses the Box-Muller algorithm to transform raw()'s into
      gaussian deviates.

      Returns:
      :   a random real with a gaussian distribution, standard deviation


    - #### gaussian

      ```
      public double gaussian(double sd)
      ```

      Parameters:
      :   `sd` - standard deviation

      Returns:
      :   a gaussian distributed random real with standard deviation
          **sd**


    - #### powlaw

      ```
      public double powlaw(double alpha,
                  double cut)
      ```

      generate a power-law distribution with exponent `alpha` and
      lower cutoff `cut`

      Parameters:
      :   `alpha` - the exponent
      :   `cut` - the lower cutoff


    - #### raw

      ```
      public abstract double raw()
      ```

      Returns a uniformly distributed pseudo-random number. The abstract method
      that must be defined to make a working RandomElement. See the class
      `RandomJava` for an example of how to do this.

      Returns:
      :   a random double in the range (0,1).

      See Also:
      :   `RandomJava`


    - #### raw

      ```
      public void raw(double[] d)
      ```

      Fill an entire array with doubles. This method calls
      `raw(double d[],int n)` with `d=d.length`. Since
      this adds little overhead for `d.length` large, it is only
      necessary to override `raw(double d[],int n)`

      Parameters:
      :   `d` - array to be filled with doubles.


    - #### raw

      ```
      public void raw(double[] d,
             int n)
      ```

      Fill part or all of an array with doubles. The method defined here uses
      multiple calls to `raw()` to fill the array. You can eliminate
      the overhead of multiple method calls by subclassing this with a version
      of the generator that fills the array. On our system this improves the
      efficiency of `Ranecu` by 20% when filling large arrays.

      Parameters:
      :   `d` - array to be filled with doubles
      :   `n` - number of doubles to generate


    - #### uniform

      ```
      public double uniform(double lo,
                   double hi)
      ```

      Parameters:
      :   `lo` - lower limit of range
      :   `hi` - upper limit of range

      Returns:
      :   a uniform random double in the range
          (**lo**,**hi**).


- Overview
- Package
- Class
- Use
- Tree
- Deprecated
- Index
- Help

- Prev Class
- Next Class

- Frames
- No Frames

- All Classes

- Summary:
- Nested |
- Field |
- Constr |
- Method

- Detail:
- Field |
- Constr |
- Method
